# Morphological and Chemical Changes in the Hemolymph of the Wax Moth *Galleria mellonella* Infected by the Entomopathogenic Fungus *Conidiobolus coronatus*

**DOI:** 10.3390/pathogens14010038

**Published:** 2025-01-07

**Authors:** Mieczysława Irena Boguś, Agata Kaczmarek, Anna Katarzyna Wrońska, Mikołaj Drozdowski, Lena Siecińska, Ewelina Mokijewska, Marek Gołębiowski

**Affiliations:** 1 Museum and Institute of Zoology, Polish Academy of Sciences, ul. Twarda 51/55, 00-818 Warszawa, Poland; akaczmarek@miiz.waw.pl (A.K.); awronska@miiz.waw.pl (A.K.W.); mikolajdrozdowski@gmail.com (M.D.); 2 BIOMIBO, ul. Strzygłowska 15, 04-872 Warszawa, Poland; lena_siecinska@wp.pl (L.S.); mokijewskaewelina@gmail.com (E.M.); 3 Laboratory of Analysis of Natural Compounds, Department of Environmental Analysis, Faculty of Chemistry, University of Gdańsk, Wita Stwosza 63, 80-308 Gdańsk, Poland; marek.golebiowski@ug.edu.pl

**Keywords:** pest, wax moth, entomopathogen, mycosis, immunocompetence, hemocytes, GC-MS

## Abstract

Hemolymph enables communication between organs in insects and ensures necessary coordination and homeostasis. Its composition can provide important information about the physiological state of an insect and can have diagnostic significance, which might be particularly important in the case of harmful insects subjected to biological control. *Galleria mellonella* Linnaeus 1758 (Lepidoptera: Pyralidae) is a global pest to honey bee colonies. The hemolymph of its larvae was examined after infection with the soil fungus *Conidiobolus coronatus* (Constantin) Batko 1964 (Entomophthorales). It was found that after one hour of contact with the fungus, the volume of the hemolymph increased while its total protein content decreased. In larvae with a high pathogen load, just before death, hemolymph volume decreased to nearly initial levels, while total protein content and synthesis (incorporation of ^35^S-labeled methionine) increased. The hemolymph polypeptide profile (SDS-PAGE followed by autoradiography) of infected insects was significantly different from that of healthy larvae. Hemocytes of infected larvae did not surround the fungal hyphae, although they encapsulated small foreign bodies (phase contrast microscopy). Infection had a negative effect on hemocytes, causing oenocyte and spherulocyte deformation, granulocyte degranulation, plasmatocyte vacuolization, and hemocyte disintegration. GC-MS analysis revealed the presence of 21 compounds in the hemolymph of control insects. *C. coronatus* infection caused the appearance of 5 fatty acids absent in healthy larvae (heptanoic, decanoic, adipic, suberic, tridecanoic), the disappearance of 4 compounds (monopalmitoylglycerol, monooleoylglycerol, monostearin, and cholesterol), and changes in the concentrations of 8 compounds. It remains an open question whether substances appearing in the hemolymph of infected insects are a product of the fungus or if they are released from the insect tissues damaged by the growing hyphae.

## 1. Introduction

The hemolymph circulating in the hemocoel of insects plays a similar role to that of blood in vertebrates. By providing organs, tissues, and cells with nutrients while also transporting harmful metabolic products to the excretory system and transporting hormones and other intercellular signaling molecules, the hemolymph enables communication between organs and ensures the necessary coordination and homeostasis within the organism; however, unlike the blood of vertebrates, the hemolymph does not participate in oxygen transport in most insect species. Also, hemolymph provides hydrostatic pressure, which allows many soft-bodied insects to maintain proper body shape, as well as the appropriate internal pressure needed in hatching, molting, and eclosion, and in the initial stretching of the wings [1,2,3].

Hemolymph is composed mainly of water and makes up 15–75% of the insect’s volume, although this varies significantly according to species, developmental stage, and individual physiological state [1,4]. In addition to inorganic compounds (mainly mineral salts), insect hemolymph contains a wealth of organic compounds. Among the carbohydrates, trehalose plays a dominant role as the energy source. In addition, it includes proteins, peptides, and amino acids that perform various functions in the body, as well as lipids, intermediate products of the Krebs cycle, uric acid, and hormones [1]. Like vertebrate blood, hemolymph contains cells involved in defensive reactions, called hemocytes, which are found as morphologically and functionally distinct subpopulations [5,6]. The immune response of insects, despite lacking adaptive capabilities, is highly effective. It comprises two equally important and closely related components: a cellular response, which includes phagocytosis, encapsulation, and nodulation processes performed by specific immune cells, and a humoral response, involving the production of antimicrobial peptides (AMPs), the activation of the prophenoloxidase (proPO) system regulating the coagulation and melanization of hemolymph, and the participation of reactive oxygen and nitrogen species [7,8,9,10,11,12,13,14,15].

It is important to limit the population size of many insect species, which could be considered pests due to their impact on human activities and the economy, to reduce economic losses and negate threats to human and animal health. One approach for controlling insect populations that has become increasingly popular is biological pest control using entomopathogenic microorganisms [16,17,18]. Therefore, it is necessary to understand, in detail, both the course of the infection process and the defense mechanisms employed by infected insects. The composition of the insect hemolymph, like that of mammalian blood, can provide a wealth of information about the physiological state of the infected organism and may have similar diagnostic significance.

The greater wax moth, *Galleria mellonella* (Pyralidae), is a serious pest to all the honey bee species (*Apis mellifera*, *A. cerana*, *A. dorsata,* and *A. florea*) colonies and occurs worldwide, causing damage to weakened hives and stored combs [19,20,21,22,23]. Due to the high susceptibility of *G. mellonella* larvae to pathogens, the great similarity between their immunity and mammalian innate immunity, and the low cost of breeding, this insect is currently widely used in laboratories as a model, allowing the reduction of the number of tests on vertebrates in toxicological studies and the examination of the innate immune response [24,25,26]. One area of particular interest concerns research on fungal infections, including those caused by fungi that are pathogenic to humans [27,28]. *Conidiobolus coronatus* (Entomophthorales) is a cosmopolitan soil fungus, a typical saprotroph known to cause mycoses in a broad spectrum of mammals and insects, including *G. mellonella* [29,30,31,32,33,34,35]. Despite numerous studies by our team on the insecticidal metabolites of this fungus and their influence on immunity in the wax moth [34,35,36,37,38,39,40,41], little is still known about the changes occurring in the hemolymph of infected insects. Therefore, the aim of the present paper was to confirm whether fungal infection is accompanied by specific hemolymph markers, changes in the composition of hemolymph, and yields characteristic changes in the hemocyte system of the invaded insect. The testable hypothesis focused on the use of *G. mellonella* as a model to study fungal infection, with the hemolymph serving as a physiological indicator for characterizing the pathogenesis. In particular, we decided to investigate how *C. coronatus* infection affects the morphotic elements of the hemolymph (hemocytes) and basic physicochemical parameters, such as hemolymph volume, total protein content, protein synthesis, polypeptide profile, and chemical composition.

## 2. Materials and Methods

### 2.1. Insects

*Galleria mellonella* larvae were reared in constant darkness at 30 °C and 70% relative humidity on an artificial diet [42]. Freshly-molted ultimate (7th) instar larvae, recognized by the white color of the cuticle and head capsule that last for two hours after molting, were collected at 24 h intervals; therefore, the age of the larvae used in the studies was determined with an accuracy of ± two hours. The body weights of untreated, normally developing larvae and infected insects were determined, together with their hemolymph volume and protein content. During normal development, *G. mellonella* larvae physiologically cease feeding on the fifth day of the last instar (5dL7) when they enter the wandering period. Formation of pupae begins on the eighth day of the final larval instar (8dL7).

### 2.2. Fungus

This study used the entomopathogenic fungus *Conidiobolus coronatus* (Entomophthorales), strain number 3491, originally isolated from *Dendrolaelaps* spp. by Professor Stanisław Bałazy (Polish Academy of Sciences, Research Center for Agricultural and Forest Environment, Poznań). The fungus was cultivated on Sabouraud agar medium supplemented with homogenized *G. mellonella* larvae to a final concentration of 10% (SAB-GM) at 20 °C and under a 12 h photoperiod (L:D 12:12) to stimulate sporulation [43]. The fungal colonies were cultured for seven days before being used to infect *G. mellonella* larvae.

### 2.3. Fungal Infection

The tested insects comprised one-day-old last-instar larvae (1dL7) and five-day-old last-instar larvae (5dL7). These were subjected to 24 h contact with sporulating fungal colonies at 20 °C. Twenty larvae were applied to each 90 mm Petri dish fully overgrown with a sporulating fungal colony. The controls consisted of larvae exposed to a sterile SAB-GM medium. Previous studies have shown that this method is the closest to the natural infection process and is the most effective [36]. Larvae were not starved and had constant access to food except for 24 h when they were exposed to the fungal colony (infected insects) or sterile SAB-GM medium (control).

The microscope observations and photo documentation of mycosed insects, their internal organs, and hemocytes were obtained using an Axio Vert.A1 fluorescence microscope (Zeiss, Jena, Germany) with Axio Cam 305 color (Zeiss, Jena, Germany) and ZEN 3.2 lite software with Modul Image Analysis (Zeiss, Jena, Germany).

### 2.4. Determination of Hemolymph Volume

This method was calibrated by measuring the uptake of tritium-labeled inulin (^3^H-inulin; 4.9 Ci/mmol; Amersham International, Whitchurch, UK) by the organs and tissues of *G. mellonella* larvae. Five-day-old last instar larvae (5dL7) were water-anesthetized (15 min in tap water at room temperature) and injected with a 1 µL solution of ^3^H-inulin dissolved in insect saline (IPS), prepared as described previously [44], containing 0.1 µCi (2.2 × 10^5^ dpm). Control insects received 1 µL of IPS.

One hour after injection, the proleg of the larva was cut off, and freely flowing hemolymph was collected on ice. A total of 5 to 20 µL of hemolymph was obtained from one larva. The collected hemolymph was centrifuged for 10 min at 5000× *g* at 2 °C in order to remove hemocytes. A total of 1 µL of serum was used to measure radioactivity and inulin dilution rate was calculated.

The body wall, fat body, digestive tract, and spinning glands of the same individuals were dissected in ice-cold IPS and briefly washed in IPS three times to remove hemolymph and attached hemocytes. The weights of all collected samples were determined. Dissected organs were homogenized in fresh ice-cold IPS, centrifuged (10 min at 5000× *g*) at 2 °C, and the radioactivity of the supernatants was measured using a Beckman Coulter (Brea, CA, USA) LS6500 liquid scintillation counter, as described for sampling and measuring tritium [45,46]. The experiment was performed in triplicate using single individuals. Since more than 85% of radioactive inulin was detected in the hemolymph (Table 1), only hemolymph was collected from inulin-injected larvae in further experiments.

### 2.5. Protein Concentration and Labeling

Protein content was measured in the hemolymph of normally developing last instar larvae (0dL7–8dL7) and infected insects (1dL7 and 5dL7). The hemolymph was collected on ice and centrifuged (10 min at 5000× *g*) at 2 °C to remove the hemocytes as described above. The protein content of the supernatants was measured with the Protein Assay (Bio-Rad, Hercules, CA, USA) following the manufacturer’s instruction. Bovine serum albumin (BSA; Merck, Darmstadt, Germany) was used as a standard.

Hemolymph proteins were subjected to in vivo labeling. The larvae received injections of 5 μCi [^35^S]-methionine (1170 Ci/mmol, DuPont, Wilmington, DE, USA) in 5 μL of IPS and incubated for three hours at 30 °C. The hemolymph was then collected on ice and centrifuged (10 min 5000× *g*) at 2 °C. The supernatant was taken and 5 μL amounts were spotted on Whatman GF/C filter paper (Whatman, Little Chalfont, UK), precipitated with 90% trichloroacetic acid (Merck, Darmstadt, Germany), washed with acetone three times (POCH, Gliwice, Poland), and counted in a Beckman Coulter (Brea, CA, USA) LS6500 liquid scintillation counter. Equal amounts of radioactive material were loaded into each slot of the gel. Hemolymph proteins were separated by one-dimensional SDS-PAGE using 15% separation gels [47]. The gels were stained with Coomassie blue, dried under a vacuum, and subjected to autoradiography on Cronex 4 films (DuPont, Wilmington, DE, USA) following the handbook [48].

### 2.6. GC-MS Analysis of Hemolymph Components

The hemolymph samples collected from the control and infected 5dL7 larvae were weighed, and their volumes were determined before freezing. Frozen samples were extracted for five minutes in 20 mL of dichloromethane (Merck, Darmstadt, Germany), and then the extracts were evaporated under nitrogen and treated to form trimethylsilyl esters (TMSs) as described previously [40]. The extracts were analyzed using a GCMS-QP2010 system with a mass detector (Shimadzu, Kyoto, Japan) using 19-methylarachidic acid (Merck, Darmstadt, Germany) as an internal standard (IS); this was selected due to its good separation from all sample components and absence in insect samples tested so far. A detailed description of the GC-MS analysis method has been published previously [40].

### 2.7. Inspection of Hemocyte Morphology

The hemocytes were subjected to morphological examination. The larvae were anesthetized in tap water at room temperature for 15 min. The rear prolegs were then cut, and the hemolymph dripped directly onto a glass slide for collection. It was observed under phase contrast using an Axio Vert.A1 microscope (Zeiss, Jena, Germany) with Axio Cam ICc 5 and ZEN 3.2 lite software (Zeiss, Jena, Germany).

### 2.8. Statistics

The Kolmogorov–Smirnov (K–S) test, skewness, and kurtosis were used to check normality. Statistical relationships were evaluated using the *t*-test and the one-way and two-way ANOVA (three independent variables) with Tukey’s post hoc test. Statistical analysis was performed using STATISTICA 6.1 software (StatSoft Polska, Kraków, Poland).

## 3. Results

### 3.1. Hemolymph Parameters in Normally-Developing G. mellonella Last Instar Larvae

Approximately 85% of tritium-labeled inulin injected into the hemocoel remained in the hemolymph and did not accumulate in any other tested organ of the larva (Table 1). Hence, by determining the dilution factor of injected tritium-labeled inulin, it was possible to confirm the changes in hemolymph volume that occur during normal development.

**Figure 1:**
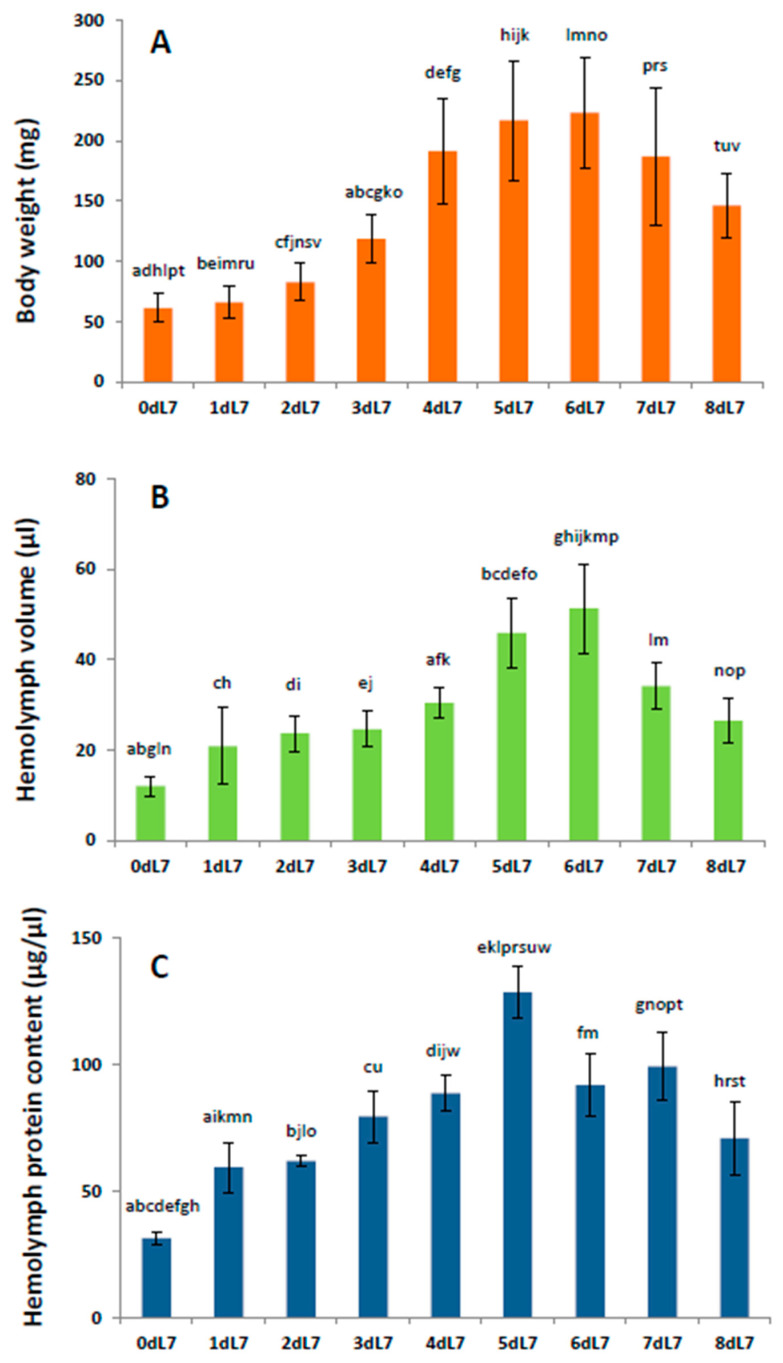
Developmental changes in the body weight (**A**), hemolymph volume (**B**), and hemolymph protein content (**C**) of the *Galleria mellonella* last instar larvae. Bars represent mean values ± standard deviation. Data statistically different (one-way ANOVA, Tukey’s post hoc test, *p* range 0.0439–0.0001) are marked with the same letter. Number of used insects: (**A**) N = 4–50, (**B**) N = 5–6, (**C**) N = 4–8. Age of insects: 0dL7—freshly molted last instar larvae (0–2 h after molting), 1dL7–8dL7 means consecutive days of the last larval instar, respectively. Formation of pupae begins on the eighth day of the final larval instar (8dL7).

The hemolymph volume of untreated, normally developing last instar larvae also reflected changes in body weight, as well as changes in the hemolymph protein content (Figure 1). The course of these changes is related to the feeding period of the last instar larvae. The physiological cessation of feeding takes place on day 5; the insects then enter a wandering period, during which they look for a place to pupate, whereupon they produce a cocoon, and pupation takes place on day 8.

### 3.2. Effects of Fungal Infection

Exposure of *G. mellonella* larvae to *C. coronatus* sporulating colonies resulted in high mortality, reaching 94 ± 5% two days later.

In the case of both 1dL7 (one-day-old, last-instar) and 5dL7 (five-day-old, last-instar) larvae, contact with the fungus caused a significant increase in hemolymph volume after just one hour, which persisted for 24 h (Table 2). However, in infected insects, just before death (48 h post-infection: 48 hpi), the hemolymph volume decreased, approaching the initial level. The total protein content in hemolymph decreased after one hour, very significantly in the case of 5dL7, but increased in both cases over the following two days. Protein synthesis, measured by the level of incorporation of ^35^S-labeled methionine, increased rapidly after one hour of contact with the fungus, especially in the case of 1dL7. In both the 1dL7 and 5dL7 larvae, the level of protein synthesis in the hemolymph was initially high but decreased slightly in the 24 hpi group and increased again just before death (48 hpi).

**Figure 2:**
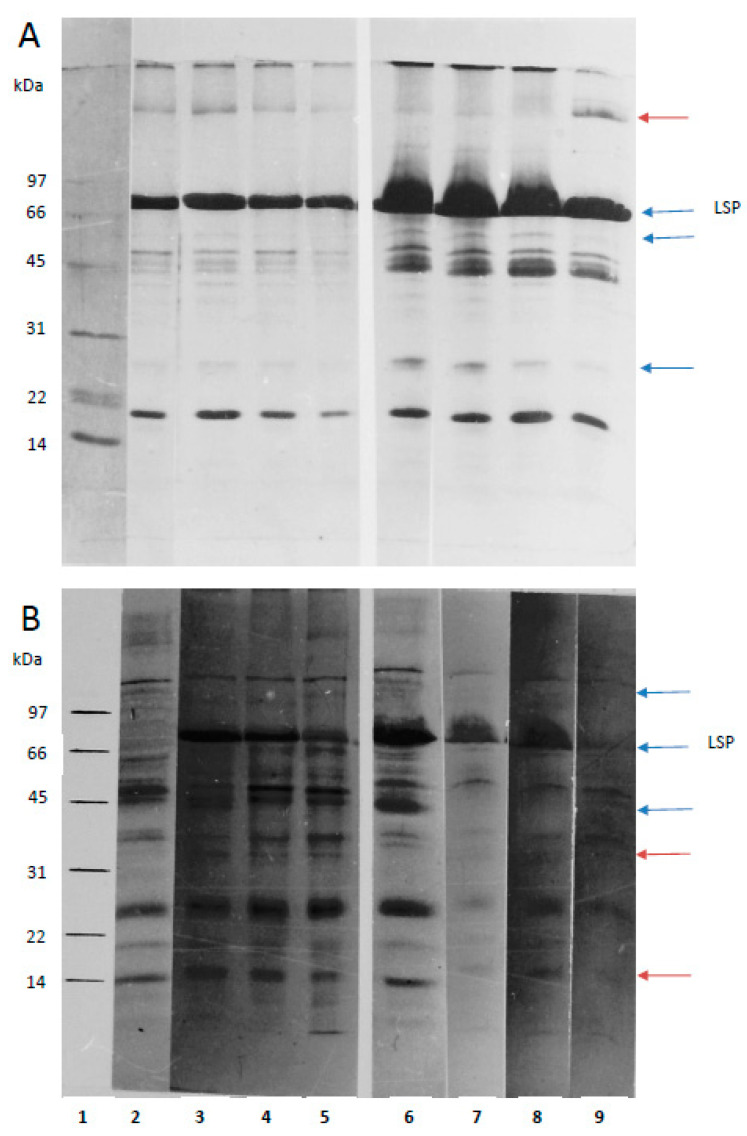
Effect of fungal infection on hemolymph protein pattern (**A**) and synthesis (**B**) in *Galleria mellonella* larvae. 1—Marker, 2—control 1dL7, 3—1dL7 1 hpi, 4—1dL7 24 hpi, 5—1dL7 48 hpi, 6—control 5dL7, 7—5dL7 1 hpi, 8—5dL7 24 hpi, 9—5dL7 48 hpi. Blue arrows indicate proteins whose concentration fluctuates during normal development, and red arrows show proteins appearing after infection. LSP—larval storage protein, hpi—hours post-infection, kDa—kilodalton.

Electrophoretic analysis (SDS-PAGE) found the untreated 1dL7 and 5dL7 to have generally similar molecular weight patterns of proteins in the hemolymph (Figure 2A); however, the 5dL7 larvae demonstrated higher levels of 74–82 kDa larval storage proteins (LPS) compared to 1dL7, and various other polypeptides with masses of approximately 26 and 45–60 kDa. Contact with the fungus caused a significant reduction in LSP content, observed in the hemolymph of dying 5dL7, as well as a reduction in 26 kDa and 60 kDa proteins and an increase in high-molecular-weight proteins (>100 kDa).

Autoradiography showed a huge diversity of polypeptides incorporating radioactively labeled methionine (Figure 2B). LSPs predominated in the hemolymph of the control 5dL7 and polypeptides of approximately 26 and 45 kDa in the control 1dL7. Interestingly, while LSP proteins with incorporated radioactive methionine were noted in infected 1dL7 larvae, these were not observed in the uninfected 1dL7. Exposure to the fungus inhibited the synthesis of a protein of approximately 45 kDa in the 5dL7 larvae, although the labeled protein was still present in the infected 1dL7 larvae. Fungal exposure also prevented the expression of a protein of around 100 kDa, which was synthesized by control 1dL7 and 5dL7. The hemolymph of the infected larvae was found to contain proteins with masses of approximately 15 and 32 kDa, which were absent from healthy insects.

**Figure 3:**
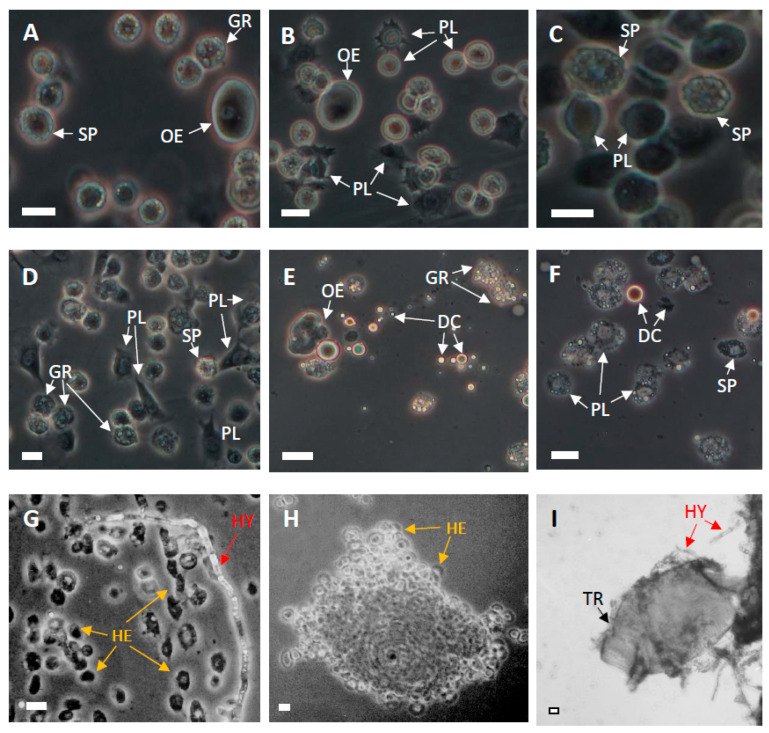
Effect of *C. coronatus* infection on *G. mellonella*: hemolymph of untreated 5dL7 controls (**A**–**C**), hemolymph of infected 5dL7 larvae (**D**–**H**), trachea from infected larva (**I**). DC—fragment of disintegrated cell, GR—granulocyte, HE—hemocyte, HY—*C. coronatus* hyphae, OE—oenocyte, PL—plasmatocyte, SP—spherulocyte, TR—trachea. Scale bars—20 μm.

Contact of 5dL7 with the fungus had a negative effect on the hemocytes circulating in the hemolymph. It also caused deformation of oenocytes and spherulocytes, degranulation of granulocytes, strong vacuolization of plasmatocytes, and disintegration of diseased hemocytes (Figure 3). In the infected insects, the fungal hyphae growing in the insect were only partially surrounded by hemocytes. However, smaller foreign bodies were still efficiently encompassed by hemocytes, which formed capsules around them (Figure 3).

The hemolymph collected from control and infected 5dL7 larvae was subjected to GC-MS analysis. The data regarding the volume of the obtained hemolymph samples, their density, and the weight of the extracts obtained from the hemolymph are given in Table 3. No significant differences in physical properties were found between hemolymph samples from healthy and infected insects; however, their chemical composition differed significantly. *C. coronatus* infection resulted in the appearance of five fatty acids, *viz.* heptanoic, decanoic, adipic, suberic, and tridecanoic acid, which were absent from healthy larvae (Table 4), as well as a reduction in monopalmitoylglycerol, monooleoylglycerol, monostearin, and cholesterol. In addition, significant decreases in the concentrations of benzoic acid, dodecanoic acid propyl ester, pentadecanoic acid, hexadecanoic acid, and octadecanoic acid were noted, accompanied by increases in those of hexadecenoic acid, heptadecenoic acid, and octadecenoic acid.

## 4. Discussion

Hemolymph volume is an important parameter for assessing the physiological state of the examined insect. It can be measured by squeezing the hemolymph from the insect or by dilution methods in which a dye (Evans Blue, Amaranth dye), or radiolabeled inulin, is injected into the hemocoel [49,50,51,52]. However, squeezing hemolymph out of the insect is a highly imprecise method associated with a high risk of underestimating the total volume, and injecting Evans Blue can overestimate the levels compared to radioactively labeled inulin by binding with other tissues [51]. Therefore, the present study used injected tritiated inulin, a 6 kDa polysaccharide; this is a reliable method that gives repeatable results; it is also non-toxic and easily soluble in body fluids [53]. Our data confirms that the vast majority of the injected inulin remained in the hemolymph during the one-hour incubation period and did not significantly accumulate in any examined organ. It should be noted that for technical reasons, the nervous, excretory, and tracheal systems, as well as the developing gonads, were omitted from this study. It cannot, therefore, be ruled out that a small part of the hemolymph surrounding the omitted organs and remaining in body compartments was not taken into account. It also cannot be ruled out that a much longer incubation may result in some of the inulin being captured from the hemolymph and sequestrated, for example, in the developing ovaries [54]. Further studies are necessary to precisely determine the error burdening our data.

In normally-developing last-instar larvae, changes in hemolymph volume have been found to follow similar patterns to changes in body mass and hemolymph protein content and are related to the period of active feeding of the larvae and the moment of its cessation before pupation; this is consistent with Shapiro’s data indicating that hemolymph volume decreased in starved *G. mellonella* larvae but continued to rise in fed larvae [55]. A similar relationship was found in *Calliphora vicina* larvae, in which the cessation of feeding and entry into the wandering stage is accompanied by a significant decrease in hemolymph volume [50]. Hemolymph volume doubled in the first four days of the last stadium in feeding *Blattella germanica* nymphs [56] and was found to rise during the latter half of an instar in *Schistocerca gregaria*, attaining its highest level just prior to ecdysis, partly due to changes in the distribution of water within the body [52].

In the *G. mellonella* larvae, exposure to *C. coronatus* resulted in infection accompanied by a significant increase in hemolymph volume. A similar effect was observed after injection with *Bacillus thuringiensis kurstaki*, i.e., decreased body water content and increased hemolymph volume [57]. Insects maintain water homeostasis by regulating the osmotic pressure of the hemolymph, with the main organs responsible for water management being the digestive tract and its accompanying Malpighian tubules. Water homeostasis is also heavily dependent on numerous transepithelial transport enablers, such as ion exchangers, pumps, co-transporters, and ion and water channels, which are subject to neurohormonal control [58,59]. The increase in hemolymph volume in infected larvae appears to occur as a result of the influx of water from the cells, probably via aquaporins: evolutionarily-conserved membrane proteins responsible for transporting water and other neutral molecules through a lipid bilayer membrane. These are present in almost all living organisms and are known to influence changes in hemocyte morphology required for cellular immune responses [60,61,62]. However, further studies are necessary to confirm the role of increased hemolymph volume in pathogen defense and the possible involvement of aquaporins in this process. The decrease in hemolymph volume observed in dying insects may be due to its exhaustion by the fungus growing in the victim’s body.

The initial decrease in total protein content noted in the hemolymph of infected insects may be due to dilution with water flowing from the cells. However, the subsequent increase in total protein concentration in heavily infected insects may be due to damage to the tissues and cells of the insect caused by the growing fungal hyphae. Increased protein synthesis just one hour after contact with the fungus may suggest the initiation of antimicrobial peptides (AMPs) synthesis; nevertheless, this would require confirmation in further studies since pathogenic fungi are known as effective immunogens that stimulate *G. mellonella* larvae to produce a broad range of AMPs [63,64,65,66].

Similarly, a decrease in protein synthesis was noted in the 24 hpi group, which may indicate a breakdown of defense processes; this was followed by another increase in synthesis just before the death of the infected insects (48 hpi group), which most likely reflects the multiplication of the fungus. The observed polypeptides with masses of 15 and 32 kDa, which are absent in the hemolymph of healthy larvae but present in infected larvae, are most likely released by the fungus growing in the host. Previous studies have shown that *C. coronatus* cultured in vitro releases a number of enzymatic proteins that hydrolyze proteins, chitin, and lipids, including a protease of 30–32 kDa [67,68,69]. Presently, however, it cannot be ruled out that these proteins constitute the insect response to infections and are part of the defensive arsenal; again, further research on this issue is essential.

Interestingly, labeled methionine was incorporated into LSP (larval storage protein) in infected 1dL7 larvae but not in control larvae. Further studies are necessary to explain this difference, as well as other discrepancies in the synthesis of various proteins between healthy and infected larvae and between larvae of different ages (1dL7 vs. 5dL7). Nevertheless, our present findings highlight the significant complexity of the response to infection.

LSP is the most abundant protein in hemolymph. It is synthesized in the fat body and secreted into the larval hemolymph; however, it is taken up by the fat body shortly before pupation [70,71]. Its role in the initiation of the defense response should also be addressed in later research, as it is known to recognize fungal PAMPs (pathogen-associated molecular patterns) [71].

It was found that while hemocytes were able to fully encapsulate smaller foreign objects, they only partially surrounded the fungal hyphae. This suggests active inhibition by the fungal hyphae, probably by the release of metabolites that inhibit the ability of hemocytes to recognize the fungus as a foreign structure or prevent adhesion to the hypha. The hemocytes also demonstrated various morphological changes, such as oenocyte and spherulocyte deformation, granulocyte degranulation, strong plasmatocyte vacuolization, and hemocyte disintegration, which are in full agreement with previous research on the effects of *C. coronatus* metabolites on immunocompetent cells [14,34,36,38,39,40,41]; these findings suggest that the growing hyphae release the same metabolites affecting hemocytes into the hemocoel as they do in vitro.

More experiments should be performed to clarify the origin of heptanoic, decanoic, adipic, suberic, and tridecanoic acids in the hemolymph of infected larvae and determine whether they derive from destroyed host cells or the growing fungus or whether they participate in the defense reaction and are actively released from immunocompetent cells. Although the fungus seems to be responsible for the disappearance of monopalmitoylglycerol, monooleoylglycerol, monostearin, and cholesterol from the hemolymph, it is not currently possible to explain the changes in the levels of other hemolymph components in the infected insects.

Changes in hemolymph volume and polypeptide and lipid profiles induced by fungal infection require further in-depth studies to explain the mechanisms underlying them. At this stage of research, it is not possible to predict whether the observed pattern of changes is unique to the *G. mellonella-C. coronatus* model or is more universal. Therefore, further studies using other fungal pathogens, as well as other host insects, are necessary. The idea of using *C. coronatus* to control wax moths in apiaries requires an indisputable demonstration of its harmlessness to bees. It is also important to remember that this fungus is capable of infecting humans and farm animals [29,30,31,32]. Therefore, it seems more reasonable to focus on the insecticidal metabolites of *C. coronatus*, several of which are approved as food additives [40,41,72].

## 5. Conclusions

Characteristic changes in key physicochemical parameters of the hemolymph of *G. mellonella* larvae caused by infection with the pathogenic soil fungus *C. coronatus* have been described. Further studies using other pathogenic fungi and insect hosts are necessary to demonstrate whether the observed changes in these parameters are universal and can be used for diagnostic purposes. The use of *C. coronatus* as a potential biological agent to control *G. mellonella* requires demonstrating the lack of negative impact of this fungus on the honeybee in both laboratory and field conditions.

## Figures and Tables

**Table 1:** Accumulation of ^3^H-inulin injected into *Galleria mellonella* larvae in various body parts.

Body Part	% of Injected Radioactivity
Body wall	9.63 ± 1.05
Fat body	0.41 ± 0.01
Digestive tract	3.05 ± 0.69
Pair of spinning glands	0.12 ± 0.01
Hemolymph	85.29 ± 2.46

Five days old last instar larvae (5dL7; N = 3) were injected with 1 µL solution of ^3^H-inulin containing 0.1 µCi (2.2 × 10^5^ dpm). One hour after injection, organs were removed, homogenized in the IPS, and centrifuged, and the radioactivity of supernatants was measured, as described in the Section 2. The experiment was performed in triplicate using single individuals (N = 3). Data are presented as means ± standard deviations. All differences between body parts in the uptake of ^3^H-inulin were statistically significant (*t*-test *p* < 0.01 or less; one-way ANOVA, the Turkey post hoc test, df = 4, F = 1777, *p* < 0.0001).

**Table 2:** Effect of *C. coronatus* infection on *G. mellonella* hemolymph volume, protein content and in vivo protein synthesis.

Treatments	Hemolymph Volume (µL)	Hemolymph Protein Content (µg/µL)	^35^S-Methionine Incorporation into Hemolymph Proteins (cpm/µL of Hemolymph)
1dL7—control	20.95 ± 8.39 ^abefg^	59.25 ± 9.84 ^ABC^	3076 ± 557 ^1,2,3,4^
1dL7—1 hpi	41.63 ± 7.14 ^achi^	55.92 ± 21.72 ^DEF^	20,499 ± 7237 ^1,5,6^
1dL7—24 hpi	58.30 ± 7.81 ^bd^	79.64 ± 21.01 ^G^	19,288 ± 1437 ^2,7,8,9^
1dL7—48 hpi	23.83 ± 7.57 ^cdjkln^	75.64 ± 23.49 ^HI^	36,380 ± 4046 ^3,10,11,12,13,14,15^
5dL7—control	45.87 ± 7.71 ^ejmo^	128.25 ± 10.31 ^ADGHJ^	8101 ± 472 ^5,7,10,11^
5dL7—1 hpi	68.17 ± 13.45 ^fhkm^	81.75 ± 8.06 ^J^	10,101 ± 1558 ^6,8,12,13^
5dL7—24 hpi	71.67 ± 13.52 ^gilop^	114.75 ± 4.72 ^BEI^	7363 ± 1850 ^9,14^
5dL7—48 hpi	47.83 ± 16.64 ^np^	111.20 ± 27.53 ^CF^	13,771 ± 542 ^4,15^

Data are presented as means ± standard deviations. 1dL7—one day old last (7th) instar larvae; 5dL7—five days old last (7th) instar larvae; hpi—hours post infection. Number of used insects: hemolymph volume N = 5–6, protein content N = 5–13, protein synthesis N = 4. Statistically significant differences (ANOVA, Tukey’s post hoc test, *p* range 0.049–0.0001) are marked with the same symbols: lowercase letters (a–p) indicate statistically significant differences in hemolymph volume, uppercase letters (A–J) indicate statistically significant differences in protein content in hemolymph, numbers (1–15) indicate statistically significant differences in hemolymph protein synthesis.

**Table 3:** Quantitative summary of the extractions: number of insects, weight and volume of collected hemolymph, masses of extracts.

	Control	Infected
Number of insects in each replication	61–80	70–78
Collected hemolymph (µL/larva)	11.05 ± 2.27	13.12 ± 1.54
Total collected hemolymph (mL)	0.74 ± 0.11	0.98 ± 0.12
Total collected hemolymph (g)	0.66 ± 0.13	0.93 ± 0.12
Density of collected hemolymph (g/mL)	0.89 ± 0.06	0.94 ± 0.02
Mass of extract (mg)	1.89 ± 1.55	1.54 ± 1.22

Hemolymph was collected from a total of 204 control and 225 infected *G. mellonella* larvae in three independent replications. Five days old last (7th) instar larvae (5dL7) were sacrificed. Infected larvae were bled 24 h post-infection (24 hpi). Data are presented as means ± standard deviations. No statistically significant differences were found between control and infected groups (ANOVA, Tukey’s test, *t*-test, *p* > 0.05).

**Table 4:** Chemical composition of the *Galleria mellonella* hemolymph collected from control and infected larvae.

Identified Compounds	Relative Content [% (*w*/*w*)]	
	Control	Infected
Hexanoic acid	0.46 ± 0.15	0.12 ± 0.08
Heptanoic acid	ND	0.01 ± 0.01
Benzoic acid	0.09 ± 0.04 *	0.01 ± 0.01 *
Octanoic acid	0.17 ± 0.08	0.04 ± 0.02
Glycerol	0.33 ± 0.24	0.18 ± 0.05
Nonanoic acid	0.30 ± 0.15	0.08 ± 0.04
Decanoic acid	ND	0.01 ± 0.01
Adipic acid	ND	0.07 ± 0.06
Dodecanoic acid, propyl ester	0.82 ± 0.27 *	0.21 ± 0.04 *
Dodecanoic acid	0.19 ± 0.06	0.18 ± 0.04
Suberic acid	ND	0.03 ± 0.04
Tridecanoic acid	ND	0.07 ± 0.04
Azelaic acid	0.14 ± 0.05	0.12 ± 0.10
Tetradecanoic acid	1.15 ± 0.03	0.93 ± 0.19
Pentadecanoic acid	1.52 ± 0.16 *	1.03 ± 0.09 *
Hexadecenoic acid	4.67 ± 0.49 **	8.46 ± 0.97 **
Hexadecanoic acid	51.72 ± 5.81 **	28.62 ± 2.79 **
Heptadecenoic acid	1.86 ± 0.18 *	4.00 ± 0.69 *
Heptadecanoic acid	0.68 ± 0.04	0.64 ± 0.05
Octadecadienoic acid	1.36 ± 0.74	1.32 ± 0.57
Octadecenoic acid	21.75 ± 5.14 **	52.56 ± 1.76 **
Octadecanoic acid	2.86 ± 0.47 *	1.33 ± 0.36 *
Monopalmitoylglycerol	1.55 ± 0.31	ND
Monooleoylglycerol	1.20 ± 0.23	ND
Monostearin	0.53 ± 0.74	ND
Cholesterol	6.67 ± 2.73	ND
Sum	100.00	100.00

Hemolymph was collected from control and infected five days old last (7th) instar larvae (5dL7) as described in the Section 2. Data are presented as means ± standard deviations of three analyses performed on separately collected and prepared samples. The number of insects used, and the data on the hemolymph samples are presented in Table 3. ND—not detected. Statistical analysis: *t*-test, * *p* < 0.05, ** *p* < 0.01.

## Data Availability

The data presented in this study are available upon request from the corresponding authors.
